# RNA-Seq data on prefrontal cortex in valproic acid model of autism and control rats

**DOI:** 10.1016/j.dib.2018.03.075

**Published:** 2018-03-21

**Authors:** Zhaoqiang Qian, Ruoxin Zhang, Jinlong Zhou, Siqi Sun, Yuanyuan Di, Wei Ren, Yingfang Tian

**Affiliations:** a Key Laboratory of Modern Teaching Technology, Ministry of Education, Xi’an, Shaanxi 710062, China; b College of Life Sciences, Shaanxi Normal University, Xi’an, Shaanxi 710119, China

## Abstract

The transcriptome sequencing data of valproic acid (VPA) model of autism and control rats are presented. VPA model of autism was induced by a single intraperitoneal injection of 600 mg/kg sodium valproic acid to female rats at day 12.5 post-conception, and the control rats were injected with saline. Male offspring of VPA- or saline-injected dams from different litters were sacrificed on PND 35 (*n* = three rats/three litters/group). Prefrontal cortex was dissected from both hemispheres, and RNA was isolated. Libraries were prepared and RNA Sequencing (RNA-Seq) was performed following Illumina's recommendations. Samples are described in the SRA portal (SRP115258) and FASTQ files have been deposited in Sequence Read Archive (accession numbers: SRR5950172 to SRR5950177). The interpretation of these data is presented in the following research article: “Transcriptional and splicing dysregulation in prefrontal cortex of valproic acid induced rat models of autism” (Zhang et al., 2018) [1].

## Specifications Table

**Table:** 

Subject area	*Biology*
More specific subject area	*Neurobiology*
Type of data	*Sequence data*
How data was acquired	*High throughput RNA sequencing*
Data format	*FASTQ files*
Experimental factors	*Valproic acid (VPA) exposure in pregnant female rats*
Experimental features	*VPA model of autism was induced by giving a single intraperitoneal injection of 600 mg/kg sodium VPA to female rats at day 12.5 post-conception, and the control rats were injected with saline.*
Data source location	*Xi’an, China*
Data accessibility	*Sample description has been deposited in the BioSample Submission Portal as Bioproject PRJNA397961and full data sets have been submitted to NCBI Sequence Read Archive (SRA,*https://www.ncbi.nlm.nih.gov/sra*) under accession numbers*SRR5950172*to*SRR5950177. *Data are publicly available.*

## Value of the data

• These data reflect the whole transcriptome of prefrontal cortex from VPA rat model of autism.
• The data could help to understand that VPA affects brain transcriptional and splicing events genome-wide.
• The data can be used to gain insight into gene-environmental interaction in autism.

## 1 Data

Data provided in this article correspond to the FASTQ files obtained after RNA sequencing of prefrontal cortex from VPA induced model of autism and control rats.

## 2 Experimental design, materials and methods

### 2.1 Experimental groups and sampling

Experimental groups and sampling as previously described [1]. Female Sprague-Dawley (SD) rats received a single intraperitoneal injection of 600 mg/kg VPA (250 mg/ml in saline, Sigma, Oakville, CA) or saline on day 12.5 post-conception. Then, the female rats were housed individually, and offspring were weaned on postnatal day (PND) 23 and separated by sex. Male offspring of VPA- or saline-injected dams from different litters were sacrificed on PND 35 (*n* = three rats/three litters/group). Dissected brain tissues were collected and frozen at − 80 °C until use.

### 2.2 RNA sequencing

RNA samples were sequenced on an Illumina Hiseq. 2000 platform (Illumina, San Diego, CA) according to the manufacturer's instructions. In brief, sequencing libraries were generated using NEBNext® Ultra™ Directional RNA Library Prep Kit for Illumina® (NEB, Ipswich, MA), and the clustering of the index-coded samples was performed on a cBot Cluster Generation System using TruSeq PE Cluster Kit v3-cBot-HS (Illumina, San Diego, CA) following manufacturer's recommendations. Then, all 6 library preparations were sequenced and 100 bp paired-end reads were generated. The full data sets have been submitted to NCBI Sequence Read Archive (SRA) under Accession.
