# Supplementary material for: RNA-Seq data on prefrontal cortex in valproic acid model of autism and control rats
Source: Data Brief. 2018 Mar 21;18:787–9. doi: 10.1016/j.dib.2018.03.075 (PMC5996313; doi:10.1016/j.dib.2018.03.075)
Supplement: Supplementary file 1 — Supplementary material [file mmc1.docx]

Manuscript ID: DIB-D-18-00294

Declarations of interest: The authors declare no conflict of interest.
